# Biopsychosocial assessment of temporomandibular disorder symptoms across bruxism subtypes in dental students: associations with stress, sleep quality, and insomnia

**DOI:** 10.22514/jofph.2026.055

**Published:** 2026-07-12

**Authors:** Merve Berika Kadıoğlu, Meyra Durmaz, Mehmet Alp Eriş, Özge Uslu-Akçam

**Affiliations:** ^1^Department of Orthodontics, Faculty of Dentistry, Ankara University, 06500 Ankara, Türkiye; ^2^Department of Oral and Maxillofacial Surgery, Faculty of Dentistry, Ankara University, 06500 Ankara, Türkiye; ^3^Department of Orthodontics, Faculty of Dentistry, Ankara Yıldırım Beyazıt University, 06220 Ankara, Türkiye

**Keywords:** Bruxism, Emotional stress, Temporomandibular disorders, Sleep health, Insomnia

## Abstract

**Background**: This study aimed to determine the prevalence of self-reported bruxism among dental students and to examine its association with stress, sleep quality, insomnia, and temporomandibular disorder (TMD) symptoms. **Methods**: A total of 480 dental students participated in the study. Based on self-reported questionnaires (subject-based assessment), participants were classified into four groups: combined bruxism, sleep bruxism, awake bruxism, and non-bruxism. Bruxism-related parameters were assessed using the Fonseca Anamnestic Index (FAI), Perceived Stress Scale-10 (PSS-10), Pittsburgh Sleep Quality Index (PSQI), and Insomnia Severity Index (ISI). Statistical analyses were performed using Pearson’s chi-square and Kruskal-Wallis tests to compare group differences, followed by multinomial logistic regression analyses to adjust for confounders. Statistical significance was defined as *p* < 0.05. **Results**: The overall prevalence of self-reported bruxism was 73.3% and was significantly higher in women (*p* = 0.001). The proportions of sleep bruxism, awake bruxism, and combined bruxism were 17.1%, 11.9%, and 44.4%, respectively. In univariate analyses, the combined bruxism group demonstrated significantly greater TMD symptom severity as measured by the FAI (*p* < 0.001; η^2^ = 0.28). In addition, perceived stress (PSS-10; *p* < 0.001; η^2^ = 0.04), sleep quality (PSQI; *p* = 0.008; η^2^ = 0.02), and insomnia severity (ISI; *p* = 0.006; η^2^ = 0.02) also differed significantly between groups; however, the corresponding effect sizes were small. In multinomial logistic regression analyses, only FAI remained independently associated with bruxism subtypes, whereas stress measures, sleep-related parameters, and demographic variables were not retained as significant predictors after adjustment. **Conclusions**: Within the limitations of a subject-based assessment, self-reported bruxism was frequently observed among dental students. Although combined bruxism was associated with higher TMD symptoms, stress, and sleep disturbances in univariate analyses, only TMD symptom severity remained independently associated after adjustment. These findings highlight the importance of assessing bruxism subtypes separately in relation to TMD symptom burden.

## 1. Introduction

Bruxism is defined by the American Academy of Sleep Medicine as repetitive jaw muscle activity characterized by clenching or grinding of the teeth [1], and it is classified into sleep bruxism (SB) and awake bruxism (AB) according to its circadian occurrence [2]. Specifically, SB involves rhythmic or non-rhythmic masticatory muscle activity during sleep, whereas AB refers to repetitive or sustained jaw muscle activity during wakefulness [3]. Although these subtypes differ in their behavioral and physiological characteristics, both have been associated with similar clinical consequences and may contribute to reduced quality of life [4].

Recurrent bruxistic activity has been associated with a range of clinical manifestations, including orofacial pain, masticatory muscle hypertrophy, tooth wear, hypersensitivity, restorative failures, and temporomandibular disorders (TMD) [5, 6, 7, 8]. However, bruxism is no longer considered solely a peripheral parafunctional habit; rather, it is increasingly conceptualized as a multifactorial motor behavior influenced by genetic, neurobiological, and psychosocial factors, including emotional stress and alterations in neurotransmitter regulation [9].

In particular, accumulating evidence has highlighted the involvement of central nervous system mechanisms in SB. Rhythmic masticatory muscle activity during sleep frequently coincides with micro-arousals and transient autonomic activation, thereby suggesting a close relationship with both sympathetic and parasympathetic nervous system modulation [10, 11]. These findings support a biopsychosocial framework for understanding the pathophysiology of bruxism.

Despite the growing body of literature, the etiology of bruxism remains incompletely understood. While divergent perspectives persist [6], a substantial body of evidence has reported associations between perceived stress and bruxistic behaviors [10, 12], and this relationship is further supported by findings of elevated salivary cortisol and catecholamine levels in individuals with bruxism [13, 14]. In addition, stress has been proposed to interact with sleep regulation mechanisms. Experimental and observational studies suggest that stress-related alterations in sleep architecture, including increased micro-arousals and instability of sleep stages, may coincide with bruxistic activity [15]. Consistent with this, recent systematic reviews have reported associations between both SB and AB and subjective sleep quality measures [16, 17]. Taken together, these findings indicate that stress and sleep parameters are closely interrelated within the biopsychosocial framework of bruxism; however, the extent to which these relationships differ across bruxism subtypes remains insufficiently clarified, particularly in populations exposed to sustained academic stress.

Dentistry, characterized by a high academic workload combined with clinical responsibilities, has consistently been described as one of the most demanding educational environments within the health sciences [18]. Given that perceived stress has been frequently examined in relation to bruxistic behaviors, dental students represent a population of particular relevance for investigation [19, 20].

Furthermore, elevated stress levels have been associated with impaired sleep quality in student populations [21]. Since sleep parameters and bruxism have been reported to interact within a broader psychophysiological framework, dental students may represent a setting in which these variables converge under sustained academic pressure [22]. Accordingly, evaluating stress- and sleep-related factors in this population is important not only for understanding oral parafunctional behaviors but also for addressing broader aspects of student well-being.

However, findings regarding the relationship between stress, sleep quality, and bruxism in dental students remain inconsistent. While some studies have reported poorer sleep quality and higher emotional distress among students with SB [23], others have identified only weak or subtype-specific associations [18, 22]. In contrast, studies employing objective assessment methods, such as polysomnography or electromyography, have failed to confirm significant associations between SB, stress, and sleep quality [12, 24].

Moreover, although the systematic review and meta-analysis by Chemelo *et al.* [4] reported an association between stress and an increased likelihood of bruxism, the overall level of evidence was considered low due to methodological heterogeneity across studies. These inconsistencies suggest that SB and AB may differ in their underlying mechanisms and therefore warrant further evaluation [12]. In addition, independent of bruxism status, significant associations have been reported between TMD, stress, and sleep quality in university students [25].

Although stress and sleep disturbances have been examined in relation to bruxism among dental students, previous studies have generally evaluated sleep and AB either collectively or separately, without systematically considering combined bruxism as a distinct analytical subgroup. Furthermore, TMD findings, perceived stress, sleep quality, and insomnia have often been investigated independently rather than concurrently within the same cohort, which limits a comprehensive understanding of their interrelationships. Consequently, evidence regarding the simultaneous evaluation of sleep, awake, and combined bruxism subtypes together with self-reported TMD symptoms, perceived stress, sleep quality, and insomnia severity within a single dental student population remains limited [18, 20, 22, 23, 25].

Therefore, the present study was designed to address this gap by examining the associations between different bruxism subtypes, including combined bruxism as a separate analytical category, and self-reported TMD symptoms, perceived stress, sleep quality, and insomnia severity using an integrated questionnaire-based approach.

The primary outcome of this study was self-reported TMD symptom severity assessed using the Fonseca Anamnestic Index (FAI), while perceived stress, sleep quality, and insomnia severity were evaluated as secondary outcomes in order to explore their associations with bruxism subtypes.

The null hypothesis of this study was that there would be no statistically significant differences among bruxism subtypes (awake, sleep, and combined) with respect to self-reported TMD symptoms, perceived stress, sleep quality, and insomnia severity in dental students.

## 2. Materials and methods

### 2.1 Study design and ethical approval

This research was conducted as a cross-sectional descriptive study at the Ankara University Faculty of Dentistry. Ethical approval was obtained from the Ankara University Ethics Committee prior to the initiation of the study (Decision No: 5/4; Date: 13 January 2025), and all procedures were performed in accordance with the principles of the Declaration of Helsinki. Furthermore, the study was designed and reported in accordance with the STROBE (Strengthening the Reporting of Observational Studies in Epidemiology) guidelines to ensure transparency and reporting consistency.

The participants were provided with detailed information regarding the purpose, scope, and potential contributions of the study, with particular emphasis placed on the principles of confidentiality, voluntariness, and anonymity. Informed consent was obtained through an online platform, and participants were informed of their right to withdraw from the study at any stage.

The data collection process was conducted solely for scientific purposes. No personal identifying information was collected, and all responses were evaluated anonymously. In addition, the research data were stored in an encrypted digital environment, with access restricted only to members of the research team.

### 2.2 Sample size and participants

The required sample size was determined based on a power analysis conducted using the study by Yıldırım *et al*. [23]. Assuming a Type I error rate (α) of 0.05, an effect size (*f*) of 0.25, and a statistical power (1 − β) of 0.90, it was estimated that a minimum total sample of at least 255 participants would be required, with at least 51 individuals in each group based on academic class.

However, recruitment was not limited to the minimum calculated sample size in order to minimize the risk of reduced statistical power due to potential data loss commonly encountered in online survey-based studies, such as incomplete responses or the exclusion of outliers. Moreover, enrolling a larger sample was intended to ensure adequate representation across different academic years and demographic subgroups within the dental student population. Accordingly, a total of 501 dental students were initially recruited. Following the exclusion of 21 individuals who reported using antidepressants or other regular medications (Fig. 1), the final analyses were conducted on 480 students (mean age: 21.21 ± 1.84 years), comprising 331 women and 149 men.

**Fig. 1.** Participant flow diagram.

The sample was selected using a convenience sampling approach and included students from all academic levels, ranging from the first to the fifth year. Dental students were selected to examine the relationships between stress, sleep parameters, and bruxism within a relatively age-homogeneous young adult population.

The inclusion criteria comprised active enrollment in dental education, being 18 years of age or older, and completion of the online questionnaire, with participation contingent upon providing informed consent. Individuals who did not provide consent were excluded from the study.

Participants were excluded if they reported conditions that could influence pain perception, muscle activity, or stress and sleep parameters, including the use of antidepressants or other psychiatric medications, the presence of diagnosed neurological or systemic disorders, or a history of botulinum toxin injections in the masticatory muscles within the last 6 months. These exclusion criteria were applied to reduce potential confounding effects on TMD symptoms, bruxism behaviors, and related psychosocial variables.

### 2.3 Data collection process

Data collection was conducted between April and May 2025 using an online survey administered through Google Forms. The survey consisted of six sections designed to assess participants’ demographic characteristics, self-reported bruxism and temporomandibular joint (TMJ) symptoms, perceived stress levels, sleep quality, and insomnia severity. Participants accessed the survey voluntarily via an invitation link and completed the questionnaire anonymously.

The survey was designed for efficient completion, with an average duration of approximately 7 minutes based on pilot testing conducted prior to data collection. It comprised brief, validated Likert-type instruments suitable for rapid self-administration. To maintain data quality, incomplete or inconsistent responses were excluded from the analysis, and all questions were configured as mandatory response fields to minimize missing data. Participants were instructed to complete the questionnaire in a single session without time pressure, and the system was configured to allow only one entry per participant in order to prevent duplicate entries. The data were collected electronically and securely archived once the target sample size had been reached.

Given the self-reported nature of the data, the potential for response bias was acknowledged. To mitigate this risk, responses were collected anonymously and completed within a single session, thereby reducing the likelihood of external influence or repeated reporting.

### 2.4 Assessments

All participants completed a structured questionnaire comprising six main sections, which together provided a comprehensive assessment of bruxism, TMD, perceived stress, sleep quality, and insomnia severity. The combined use of these instruments allowed for an integrated evaluation of the relationships between bruxism subtypes and both orofacial symptoms and the psychosocial burden related to stress and sleep.

The first section collected demographic information, including gender, academic year, lifestyle habits, and systemic health status. The second section assessed the presence of bruxism and its subtypes (sleep, awake, or combined) through self-reported questions, in accordance with the international diagnostic criteria proposed by Lobbezoo *et al*. [3]. The third section evaluated TMJ dysfunction symptoms using the FAI. The fourth section assessed perceived stress levels over the previous month using the Perceived Stress Scale-10 (PSS-10). The fifth and sixth sections evaluated sleep quality and insomnia severity using the Pittsburgh Sleep Quality Index (PSQI) and the Insomnia Severity Index (ISI), respectively.

#### 2.4.1 Demographic information form

Participants were asked to provide information on age, gender, body mass index (BMI), tobacco and alcohol use, regular medication use, history of systemic or rheumatic disease, and any application of botulinum toxin to the masticatory muscles within the preceding six months.

#### 2.4.2 Self-reported bruxism assessment

Bruxism is classified according to the method used to identify its presence. In the 2018 international consensus, bruxism was categorized as possible, probable, or definite based on the level of diagnostic evidence supporting its identification [26]. Within this framework, cases identified solely through self-report correspond to the level of possible bruxism, whereas confirmation through clinical examination and/or instrumental assessment increases diagnostic certainty to the probable or definite levels. In the updated 2025 international consensus, this hierarchical terminology was revised and replaced with the descriptors subject-based, clinically based, and device-based assessments in order to improve conceptual clarity and reduce diagnostic ambiguity [27].

In the present study, bruxism was assessed exclusively through self-report. Accordingly, the identified cases correspond to the level of possible bruxism as defined by the 2018 consensus and, under the updated terminology, represent a subject-based assessment. The self-reported bruxism questionnaire was adapted from the methodology described by Phuong *et al*. [20], whose items were derived from the American Academy of Sleep Medicine recommendations for possible SB and from questions developed by Pintado *et al.* [28] for possible AB. The questionnaire included items addressing nocturnal bruxism-related behaviors and symptoms, such as reports of tooth grinding during sleep, morning jaw discomfort, and jaw muscle fatigue upon awakening, as well as items related to daytime parafunctional activities, including conscious or unconscious clenching and grinding.

Participants who responded “Yes” to question 1 and/or 2, or to at least one symptom in question 3, were classified as having SB. Those who responded “Yes” to question 4 and/or 5 were classified as having AB. The assessment of AB in this study was limited to self-reported daytime grinding and clenching behaviors, and did not include specific behaviors such as mandibular thrusting or sustained jaw bracing without tooth contact. Therefore, the operational definition of AB in the present study reflects only the behaviors captured by the questionnaire items. Participants endorsing both SB and AB items were classified as combined bruxism, whereas those responding “No” to all items were assigned to the non-bruxism group. This classification constituted the basis of the study groups. The complete questionnaire is provided in the **Supplementary material**.

#### 2.4.3 Fonseca anamnestic index (FAI)

The FAI, originally developed by Fonseca *et al*. [29] (1994) to evaluate TMJ dysfunction, consists of 10 items assessing TMJ pain and symptoms related to the head and neck region. The Turkish version of the scale has been validated by Kaynak *et al*. [30], demonstrating that it is a reliable and valid screening tool for TMJ disorders in the Turkish population (Cronbach’s α = 0.805). The questionnaire encompasses multidimensional parameters, including joint and head-neck pain, limitations in jaw movement, parafunctional habits, occlusal problems, and emotional stress. Responses are recorded using a three-point scale: “Yes” (10 points), “Sometimes” (5 points), and “No” (0 points). Based on the total score, self-reported TMD symptom severity is categorized as follows: 0–15 = no TMD, 20–40 = mild TMD, 45–65 = moderate TMD, and 70–100 = severe TMD [31].

The FAI was selected as a screening instrument to assess self-reported TMD symptoms in a large, survey-based student sample. Although the DC/TMD (Diagnostic Criteria for Temporomandibular Disorders) is considered the reference standard for the clinical diagnosis of TMD, its application requires a structured clinical examination performed by calibrated examiners. Given the cross-sectional design and the online data collection approach of the present study, the implementation of a clinical diagnostic protocol such as the DC/TMD was not feasible. Therefore, a validated self-report screening instrument was employed to enable standardized assessment within the study framework.

#### 2.4.4 Perceived stress scale-10 (PSS-10)

The Perceived Stress Scale (PSS), one of the most widely used instruments for the subjective assessment of stress levels, was originally developed by Cohen *et al.* [32] (1983). The Turkish version of the scale was validated by Eskin and demonstrated high internal consistency (Cronbach’s α = 0.82) [33].

The PSS evaluates individuals’ perceived stress by assessing their thoughts and feelings over the preceding month. Each item is scored on a 5-point Likert scale ranging from 0 (never) to 4 (very often), and the total score is used to classify stress levels, with scores of 0–13 indicating low stress, 14–26 indicating moderate stress, and scores ≥27 indicating high stress [34]. The 10-item version of the scale (PSS-10) was preferred in the present study due to its superior psychometric reliability compared to the original 14-item form [35].

#### 2.4.5 Pittsburgh sleep quality index (PSQI)

PSQI is a self-report instrument developed by Buysse *et al.* [36] (1989) to assess sleep quality and sleep-related disturbances over the past month. The Turkish validity and reliability of the PSQI were established by Ağargün *et al*. [37], demonstrating that the scale can be reliably applied within the Turkish population. The PSQI consists of 19 items and evaluates seven components, including subjective sleep quality, sleep latency, sleep duration, habitual sleep efficiency, sleep disturbances, use of sleep medication, and daytime dysfunction. A PSQI score >5 was determined as indicative of poor sleep quality [35].

#### 2.4.6 Insomnia severity index (ISI)

ISI is a reliable, self-report, seven-item instrument that assesses insomnia symptoms and sleep-related difficulties over the past two weeks [38]. The scale evaluates multiple domains, including difficulty initiating sleep, difficulty maintaining sleep, early morning awakening, dissatisfaction with sleep quality, interference with daily functioning, and the degree of distress associated with sleep problems. The Turkish version of the ISI was validated by Boysan *et al.* [39] (2010), demonstrating high internal consistency (Cronbach’s α = 0.79). Total scores are categorized as follows: 0–7 (absence of insomnia), 8–14 (subthreshold insomnia), 15–21 (moderate insomnia), and 22–28 (severe insomnia) [40].

Both the PSQI and ISI were included in the present study to capture complementary dimensions of sleep-related functioning. While the PSQI provides a multidimensional assessment of overall sleep quality over the past month, the ISI focuses specifically on the perceived severity and daytime impact of insomnia symptoms over a shorter timeframe. The combined use of these instruments allowed for a broader and more nuanced characterization of sleep-related complaints within the study population.

### 2.5 Statistical analysis

Statistical analyses were performed using IBM SPSS Statistics for Windows, Version 22.0 (IBM Corp., Armonk, NY, USA). Descriptive statistics were expressed as frequencies and percentages for categorical variables and as median (minimum–maximum) values for continuous variables. Comparisons of categorical variables between groups were conducted using the Pearson chi-square test. For continuous or ordinal variables involving comparisons across more than two groups, the Kruskal-Wallis H test was applied, as the assumptions for parametric testing were not met. When a statistically significant difference was identified using the Kruskal-Wallis test, pairwise comparisons were subsequently performed using Dunn’s *post-hoc* test, with Bonferroni correction applied to account for multiple comparisons and to control the risk of Type I error. In cases where the assumptions of the chi-square test were not satisfied, Monte Carlo correction was employed. Effect sizes for group comparisons were calculated and reported as eta squared (η^2^) for the Kruskal-Wallis test to quantify the magnitude of observed differences. The interpretation of effect sizes followed Cohen’s criteria, whereby η^2^ = 0.01 indicates a small effect, η^2^ = 0.06 a medium effect, and η^2^ ≥ 0.14 a large effect [41]. To control for potential confounding variables, multinomial logistic regression analyses were conducted to identify independent predictors of bruxism subtypes. Sex, smoking status, BMI, academic year, perceived stress (PSS-10), sleep quality (PSQI), and insomnia severity (ISI) were entered into the model as covariates. Due to the limited number of participants in the obesity category, overweight and obesity groups were combined in the regression analysis to improve model stability. Prior to conducting regression analyses, multicollinearity among continuous predictors was assessed using tolerance values and variance inflation factor (VIF) statistics. Adjusted odds ratios (ORs) with 95% confidence intervals (CIs) were reported. All statistical analyses were two-tailed, and the level of statistical significance was set at α = 0.05.

## 3. Results

A total of 44.38% of participants (n = 213) were classified as having combined bruxism, 17.08% (n = 82) were categorized as SB, 11.87% (n = 57) as AB, and 26.67% (n = 128) as non-bruxism (Table 1).

**Table 1.** Distribution of study groups according to bruxism type.

Study Groups	n	%
Combined bruxism	213	44.38
Sleep bruxism	82	17.08
Awake bruxism	57	11.87
Non-bruxism	128	26.67

*n: number of participants.*

Regarding demographic characteristics, 69% of participants were female, and 31% were male. A statistically significant association was identified between gender and bruxism prevalence, with a higher proportion of combined bruxism observed among female students (Table 2).

**Table 2.** Demographic characteristics of participants according to bruxism type.

Variables		Combined Bruxism		Sleep Bruxism		Awake Bruxism		Non-Bruxism		Total		Chi-Square Analysis	
		n	%	n	%	n	%	n	%	n	%	χ^2^	*p*-value
Gender													
	Female	164	77.0	56	68.3	35	61.4	76	59.4	331	69.0	13.4	**0.001**
	Male	49	23.0	26	31.7	22	38.6	52	40.6	149	31.0		
	Total	213	100.0	82	100.0	57	100.0	128	100.0	480	100.0		
Grade													
	1st year	28	13.1	14	17.1	11	19.3	25	19.5	78	16.3	10.3	0.583
	2nd year	48	22.5	21	25.6	12	21.1	33	25.8	114	23.8		
	3rd year	54	25.4	21	25.6	18	31.6	31	24.2	124	25.8		
	4th year	37	17.4	11	13.4	9	15.8	24	18.8	81	16.9		
	5th year	46	21.6	15	18.3	7	12.3	15	11.7	83	17.3		
	Total	213	100.0	82	100.0	57	100.0	128	100.0	480	100.0		
Body Mass Index													
	Underweight	23	10.8	7	8.5	5	8.8	11	8.6	46	9.6	5.7	0.765
	Normal	160	75.1	57	69.5	41	71.9	91	71.1	349	72.7		
	Overweight	27	12.7	14	17.1	10	17.5	22	17.2	73	15.2		
	Obesity	3	1.4	4	4.9	1	1.8	4	3.1	12	2.5		
	Total	213	100.0	82	100.0	57	100.0	128	100.0	480	100.0		
Smoking													
	Yes	68	31.9	14	17.1	18	31.6	27	21.1	127	26.5	9.6	**0.022**
	No	145	68.1	68	82.9	39	68.4	101	78.9	353	73.5		
	Total	213	100.0	82	100.0	57	100.0	128	100.0	480	100.0		
Alcohol Consumption													
	Yes	77	36.2	22	26.8	20	35.1	36	28.1	155	32.3	3.7	0.285
	No	136	63.8	60	73.2	37	64.9	92	71.9	325	67.7		
	Total	213	100.0	82	100.0	57	100.0	128	100.0	480	100.0		

*n: number of participants; χ^2^: Chi-square value. Bold values indicate statistically significant differences (p < 0.05).*

No statistically significant associations were found between bruxism status and educational period, BMI, or alcohol consumption (*p* > 0.05). In contrast, smoking status differed significantly across bruxism groups, with higher smoking rates observed in the awake and combined bruxism groups (*p* = 0.022) (Table 2).

TMD (FAI), perceived stress (PSS-10), sleep quality (PSQI), and insomnia severity (ISI) were evaluated using both categorical distributions (Table 3) and continuous measures (Table 4), and group comparisons demonstrated significant differences across bruxism subtypes.

**Table 3.** Categorical distribution of bruxism groups according to temporomandibular disorder (FAI), stress level (PSS-10), sleep quality (PSQI), and insomnia level (ISI).

Variables		Combined Bruxism		Sleep Bruxism		Awake Bruxism		Non-Bruxism		Total		Chi-Square Analysis	
		n	%	n	%	n	%	n	%	n	%	χ^2^	*p*-value
Fonseca Anamnestic Index (FAI)													
	No TMD	16	13.2	21	17.4	21	17.4	63	52.1	121	100.0	137.6	**0.0001**
	Mild TMD	72	37.3	37	19.2	29	15.0	55	28.5	193	100.0		
	Moderate TMD	80	70.8	17	15.0	6	5.3	10	8.8	113	100.0		
	Severe TMD	45	84.9	7	13.2	1	1.9	0	0.0	53	100.0		
	Total	213	44.4	82	17.1	57	11.9	128	26.7	480	100.0		
Perceived Stress Scale-10 (PSS-10)													
	Low Stress	5	22.7	4	18.2	4	18.2	9	40.9	22	100.0	11.5	0.074
	Moderate Stress	168	43.2	66	17.0	47	12.1	108	27.8	389	100.0		
	High Stress	40	58.0	12	17.4	6	8.7	11	15.9	69	100.0		
	Total	213	44.4	82	17.1	57	11.9	128	26.7	480	100.0		
Pittsburgh Sleep Quality Index (PSQI)													
	Good Sleep	48	35.6	22	16.3	26	19.3	39	28.9	135	100.0	12.3	**0.006**
	Poor Sleep	165	47.8	60	17.4	31	9.0	89	25.8	345	100.0		
	Total	213	44.4	82	17.1	57	11.9	128	26.7	480	100.0		
Insomnia Severity Index (ISI)													
	Absence	61	39.9	21	13.7	26	17.0	45	29.4	153	100.0	162*	**0.047**
	Sub-Threshold	108	43.4	47	18.9	28	11.2	66	26.5	249	100.0		
	Moderate	41	59.4	11	15.9	3	4.3	14	20.3	69	100.0		
	Severe	3	33.3	3	33.3	0	0.0	3	33.3	9	100.0		
	Total	213	44.4	82	17.1	57	11.9	128	26.7	480	100.0		

n: number of participants; χ^2^: Chi-square value; p: statistical significance; FAI: Fonseca An-amnestic Index; PSS-10: Perceived Stress Scale-10; PSQI: Pittsburgh Sleep Quality Index; ISI: Insomnia Severity Index; TMD: temporomandibular disorder. Data are presented as frequency (n) and percentage (%). *Monte Carlo correction. Bold values indicate statistically significant differences (p < 0.05).

**Table 4.** Comparison of total scores of Fonseca Anamnestic Index, Perceived Stress Scale-10, Pittsburgh Sleep Quality Index, and Insomnia Severity Index among bruxism groups.

Variables	Combined Bruxism (1)	Sleep Bruxism (2)	Awake Bruxism (3)	Non-Bruxism (4)	Total	Kruskal-Wallis H Test			
	Mean ± SD (Min–Max)	Mean ± SD (Min–Max)	Mean ± SD (Min–Max)	Mean ± SD (Min–Max)	Mean ± SD (Min–Max)	H	*p*-value	*Post-hoc*	η^2^
Fonseca Anamnestic Index (FAI)	48.00 ± 22.15 (0–100)	32.68 ± 21.59 (0–80)	25.44 ± 17.28 (0–70)	19.18 ± 15.80 (0–65)	35.02 ± 23.42 (0–100)	137.8	**<0.001**	1–2, 1–3, 1–4, 2–4	0.28
Perceived Stress Scale (PSS-10)	22.13 ± 5.17 (6–38)	20.50 ± 5.29 (3–34)	19.63 ± 4.90 (7–30)	19.88 ± 5.29 (0–40)	20.96 ± 5.29 (0–40)	22.03	**<0.001**	1–3, 1–4	0.04
Pittsburgh Sleep Quality Index (PSQI)	8.19 ± 3.38 (0–18)	7.72 ± 3.58 (1–19)	6.54 ± 2.98 (2–13)	7.41 ± 3.53 (0–17)	7.71 ± 3.44 (0–19)	11.07	**0.008**	1–2, 1–3, 1–4	0.02
Insomnia Severity Index (ISI)	10.50 ± 4.77 (0–25)	10.50 ± 4.86 (0–24)	8.44 ± 3.56 (1–17)	9.33 ± 4.93 (0–23)	9.94 ± 4.75 (0–25)	12.5	**0.006**	1–3, 1–4, 2–3, 2–4	0.02

*SD: standard deviation; Min–Max: Minimum–maximum; H: Kruskal-Wallis test statistic; p: statistical significance; η^2^: Eta-square effect size. Data are presented as Mean ± SD (Min–Max). Comparisons among the four bruxism groups were performed using the Kruskal-Wallis H test followed by Dunn-Bonferroni post hoc analysis. Bold values indicate statistically significant differences (p < 0.05).*

Analysis of FAI revealed a significant relationship between TMD severity and bruxism status (χ^2^ = 137.6; *p* < 0.001) (Table 3, Fig. 2). Notably, the majority of moderate and severe TMD cases in the combined bruxism group accounted for 70.8% and 84.9% of these categories, respectively, whereas TMD was predominantly absent or classified as mild in the non-bruxism group.

**Fig. 2.** Distribution of participants according to bruxism type (combined, sleep, awake, and non-bruxism). (A) Self-reported TMD symptom severity based on the Fonseca Anamnestic Index (FAI); (B) perceived stress level based on the PSS-10; (C) sleep quality based on the Pittsburgh Sleep Quality Index (PSQI); and (D) insomnia severity based on the Insomnia Severity Index (ISI). Each column represents the percentage distribution of participants within each bruxism group, normalized to 100%. Percentages were calculated relative to the total number of participants in each group. TMD: temporomandibular disorder.

In contrast, analysis of the PSS-10 categorical stress levels did not demonstrate a statistically significant difference across bruxism groups (*p* = 0.074) (Table 3). Although a numerically higher proportion of individuals with high perceived stress was observed in the combined bruxism group, this pattern did not reach statistical significance and should therefore be interpreted with caution (Fig. 2).

The PSQI results demonstrated a statistically significant association between sleep quality and bruxism type (χ^2^ = 12.3; *p* = 0.006) (Table 3). The highest proportion of poor sleep quality was observed in the combined bruxism group (47.8%). In contrast, within the AB group, 26 of 57 participants were classified as having good sleep quality (Table 3).

Analysis of the ISI also revealed statistically significant differences in insomnia levels across bruxism groups (*p* = 0.047) (Table 3). Most participants were categorized within the subthreshold insomnia group (n = 249). Among those with moderate clinical insomnia, 59.4% were classified within the combined bruxism group, whereas severe insomnia was observed at low frequencies across all groups (Table 3).

When continuous total scores of the FAI, PSS-10, PSQI, and ISI were compared across bruxism subtypes, statistically significant differences were identified for all parameters (*p* < 0.05) (Table 4).

According to the FAI results (Table 4), a significant difference was observed among the groups in terms of TMD severity (H = 137.8; *p* < 0.001). *Post-hoc* analysis demonstrated that the mean FAI score of the combined bruxism group was significantly higher than those of all other groups. In addition, a significant difference was identified between the SB and non-bruxism groups. These findings indicate that TMD symptoms were most pronounced in the combined bruxism group (48.00 ± 22.15) and lowest in the non-bruxism group (19.18 ± 15.80). The magnitude of this difference corresponded to a large effect size (η^2^ = 0.28).

A statistically significant difference was also observed among bruxism groups in the Perceived Stress Scale (PSS-10) total scores (H = 22.03; *p* < 0.001) (Table 4). This difference was mainly attributable to higher stress levels in the combined bruxism group compared to both the AB and non-bruxism groups. The magnitude of this difference was small to moderate (η^2^ = 0.04).

Notably, while categorical PSS-10 stress levels did not differ significantly across bruxism groups (χ^2^ = 11.5; *p* = 0.074) (Table 3), analysis of continuous total PSS-10 scores revealed a statistically significant difference (H = 22.03; *p *< 0.001) (Table 4). *Post-hoc* comparisons indicated that participants in the combined bruxism group had higher total PSS-10 scores than those in the AB and non-bruxism groups.

With respect to sleep quality, analysis of PSQI total scores demonstrated a statistically significant difference among bruxism groups (H = 11.07; *p* = 0.008) (Table 4). *Post-hoc* analysis indicated that the combined bruxism group had significantly higher PSQI scores compared to the SB, AB, and non-bruxism groups. The magnitude of this difference was small (η^2^ = 0.02).

Similarly, ISI total scores differed significantly across bruxism groups (H = 12.5; *p* = 0.006) (Table 4). *Post-hoc* analysis showed that both the combined bruxism and SB groups had significantly higher ISI scores compared to the AB and non-bruxism groups. The observed differences were of small magnitude (η^2^ = 0.02).

Before performing the multinomial logistic regression analysis, multicollinearity among the continuous predictors was assessed using tolerance and VIF statistics. The tolerance values were 0.846 for FAI, 0.776 for PSS-10, 0.567 for PSQI, and 0.531 for ISI, and the corresponding VIF values were 1.182, 1.289, 1.765, and 1.883, respectively. As all tolerance values exceeded 0.10 and all VIF values were below 10, no evidence of problematic multicollinearity was identified, indicating that the variables were suitable for inclusion in the regression model. In addition, due to the limited number of participants in the obesity category, overweight and obesity groups were combined in the multinomial regression analysis to improve model stability.

Multinomial logistic regression analysis demonstrated that higher FAI scores were significantly associated with increased odds of all bruxism subtypes (Table 5), including combined bruxism (OR = 1.08, 95% CI: 1.06–1.10, *p* < 0.001), SB (OR = 1.04, 95% CI: 1.03–1.06, *p* < 0.001), and AB (OR = 1.03, 95% CI: 1.01–1.05, *p* = 0.008). In contrast, perceived stress (PSS-10), sleep quality (PSQI), and insomnia severity (ISI) were not independently associated with bruxism subtypes after adjustment for covariates (all *p* > 0.05). Similarly, demographic and lifestyle variables, including sex, smoking status, BMI categories, and academic year, were not independently associated with any bruxism subtype in the adjusted model (Table 5).

**Table 5.** Multinomial logistic regression analysis examining independent predictors of bruxism subtypes.

Variables		Combined Bruxism		Sleep Bruxism		Awake Bruxism	
		*p*-value	OR (95% CI)	*p*-value	OR (95% CI)	*p*-value	OR (95% CI)
FAI		**<0.001**	1.08 (1.06–1.10)	**<0.001**	1.04 (1.03–1.06)	**0.008**	1.03 (1.01–1.05)
PSS-10		0.527	1.02 (0.96–1.08)	0.393	0.97 (0.91–1.04)	0.945	1.00 (0.93–1.07)
PSQI		0.231	0.94 (0.85–1.04)	0.250	0.94 (0.83–1.05)	0.115	0.90 (0.80–1.03)
ISI		0.590	0.98 (0.91–1.06)	0.140	1.07 (0.98–1.16)	0.452	0.96 (0.87–1.07)
Sex (reference: Female)							
	Male	0.522	1.21 (0.67–2.20)	0.535	1.23 (0.64–2.37)	0.809	1.09 (0.54–2.19)
Smoking (reference: No)							
	Yes	0.139	0.62 (0.33–1.17)	0.338	1.45 (0.68–3.10)	0.069	0.50 (0.24–1.05)
BMI (reference: Overweight/obesity)							
	Underweight	0.542	1.40 (0.48–4.13)	0.496	0.66 (0.19–2.21)	0.896	0.91 (0.24–3.52)
	Normal	0.923	1.04 (0.51–2.10)	0.248	0.65 (0.31–1.36)	0.971	0.98 (0.43–2.27)
Academic year (reference: 5th year)							
	1st	0.146	0.50 (0.20–1.27)	0.275	0.57 (0.20–1.59)	0.945	1.04 (0.32–3.37)
	2nd	0.237	0.59 (0.25–1.41)	0.483	0.71 (0.28–1.80)	0.764	0.84 (0.27–2.61)
	3rd	0.640	0.81 (0.34–1.94)	0.658	0.81 (0.31–2.10)	0.525	1.43 (0.48–4.27)
	4th	0.951	1.03 (0.40–2.63)	0.471	0.68 (0.23–1.99)	0.965	1.03 (0.30–3.53)

*OR: Odds Ratio; CI: Confidence Interval; FAI: Fonseca Anamnestic Index; PSS-10: Perceived Stress Scale-10; PSQI: Pittsburgh Sleep Quality Index; ISI: Insomnia Severity Index; BMI: body mass index. Outcome reference category: non-bruxism. Reference categories for predictors: female sex, non-smoker, obesity (BMI), and 5th academic year. All p-values are two-tailed; statistical significance was set at p < 0.05. Bold values indicate statistically significant differences (p < 0.05).*

## 4. Discussion

This study examined the relationship between different types of bruxism (combined, SB, and AB) and TMD severity, perceived stress level, sleep quality, and insomnia severity in dental students. Given the multifactorial nature of bruxism, evaluating these variables through both psychophysiological and behavioral components is essential for a more comprehensive understanding of its etiopathogenesis. In particular, the tendency for high stress, sleep disturbances, and musculoskeletal symptoms to co-occur in young adults exposed to sustained academic demands further emphasizes the clinical relevance of bruxism in this population. Accordingly, bruxism should be considered not only as a parafunctional activity affecting orofacial structures, but also as a psychophysiological response pattern that may reflect aspects of student mental health and academic functioning.

The findings of the present study revealed that bruxism subtypes were significantly associated with self-reported psychosocial and sleep-related measures, and higher levels of TMD symptoms, perceived stress, and sleep-related complaints were observed in the combined bruxism group compared to the other subtypes. These observations are generally consistent with existing literature; however, they should be interpreted with appropriate caution, particularly in light of the subsequent multivariable analyses. While the univariate findings suggest that bruxism may be related to multiple psychosocial and sleep-related dimensions, the results also support the perspective that bruxism should not be interpreted as an isolated oral parafunction, but rather as a multidimensional condition influenced by interacting behavioral and psychophysiological factors.

The prevalence of bruxism in the general adult population has been reported to vary considerably across studies, with meta-analytic data indicating substantial variability in both SB and AB worldwide [5, 42]. In the present study, the frequency of self-reported possible bruxism among dental students appeared markedly higher than rates typically reported in broader young adult populations. This finding requires careful interpretation, as bruxism was assessed exclusively through self-report, corresponding to the “possible bruxism” level defined in the 2018 consensus and aligned with the subject-based assessment framework described in the updated 2025 International Consensus on Bruxism. In the absence of clinical examination or instrumental confirmation, such as electromyography or polysomnography, prevalence estimates may be influenced by recall bias, misclassification, and potential overestimation.

Previous studies conducted in dental student populations have also reported considerable variability in bruxism prevalence [6, 18, 19, 23, 43], highlighting the influence of methodological differences, diagnostic criteria, and reliance on self-report instruments. In addition, contextual factors specific to dental education, including high academic workload, clinical performance requirements, and evaluation-related stress, may contribute to increased reporting of bruxism-related behaviors. Therefore, the relatively elevated prevalence observed in the present study should be interpreted primarily in relation to methodological heterogeneity and the use of subject-based assessment methods. Given that this level of assessment carries a recognized risk of misclassification and overestimation, it may partly explain the discrepancy between the present findings and lower prevalence rates reported in studies using clinical or instrumental diagnostic approaches.

Studies that have evaluated sleep and AB separately in dental student populations have consistently demonstrated variability in prevalence estimates [18, 20, 22]. In addition, the proportion of individuals presenting with both sleep and AB has also varied across studies [18, 20], which may reflect differences in diagnostic criteria, reliance on self-report measures, and sample characteristics. Importantly, sleep and AB are not mutually exclusive phenomena and may co-occur within the same individual, potentially influencing one another [44]. In the present study, the distribution pattern of sleep and AB was generally consistent with previous reports; however, the overall frequencies appeared higher. As noted previously, this observation should be interpreted in the context of the self-report-based classification approach and the methodological heterogeneity observed across studies.

When demographic variables were considered, bruxism subtypes differed primarily according to gender and smoking status, whereas no meaningful differences were observed with respect to academic year, BMI, or alcohol consumption. Female students, in particular, were more frequently represented in the bruxism groups compared to males. This pattern is consistent with previous reports indicating a higher prevalence of bruxism among women. The observed gender difference may reflect sex-related variations in stress perception, coping strategies, pain sensitivity, and underlying neurobiological mechanisms; therefore, gender-specific biopsychosocial factors should be taken into account when interpreting bruxism-related findings in young adult populations [23, 45]. This observation may be further explained by increased sensitivity to stress and anxiety in women, differences in coping styles, and potential hormonal and neurobiological influences [46]. Furthermore, women’s greater susceptibility to sleep disturbances and heightened awareness of pain may contribute to the physiological basis of this difference [47]. Taken together, these findings suggest that not only environmental or academic stressors, but also gender-specific biopsychosocial mechanisms, may play a role in the development of bruxism [48].

FAI is a widely used and cost-effective screening instrument for assessing self-reported TMD symptom severity in non-clinical populations [49]. In the present study, a validated version of the FAI was employed to evaluate self-reported TMD symptoms among dental students [50]. Previous studies have reported considerable variability in FAI-based prevalence estimates within university populations [25, 51], and in line with these findings, a substantial proportion of participants in the current sample reported TMD-related symptoms. This observation supports the notion that young adult student populations may exhibit a relatively high burden of self-reported TMD complaints.

When examined according to self-reported TMD symptom severity, significant differences were observed between bruxism subtypes. Combined bruxism was associated with higher FAI scores, whereas the non-bruxism group demonstrated lower symptom levels, and AB was characterized by comparatively milder profiles. *Post-hoc* analyses further indicated that combined bruxism was associated with higher TMJ symptom scores than isolated sleep or AB, and that self-reported TMD symptom severity was greater in the SB group compared to the non-bruxism group. These findings suggest that the coexistence of SB and AB behaviors may be associated with a greater overall symptom burden, possibly reflecting cumulative mechanical loading of the masticatory system [52].

Given the relatively high proportion of combined bruxism observed in the present sample, this category should not be considered homogeneous. Variability in the predominance of sleep- versus awake-related behaviors within this group may partly account for the magnitude of the observed associations, as individuals with predominantly sleep-related activity may differ clinically from those exhibiting primarily daytime clenching behaviors. Accordingly, future studies that distinguish between SB-dominant and AB-dominant patterns within combined bruxism are warranted.

Importantly, in the multivariable logistic regression analyses, the association between bruxism subtype and self-reported TMD symptom severity remained robust even after adjustment for potential confounders. This finding suggests that the observed relationship may not be solely attributable to demographic or psychosocial factors, but may reflect a more direct association between bruxism-related behaviors and TMD symptom burden.

However, these findings should be interpreted with caution. In the present study, both bruxism classification and TMD symptoms were assessed exclusively using self-reported questionnaires. Notably, some items included in the FAI capture parafunctional behaviors and psychosocial complaints that may conceptually overlap with bruxism-related behaviors. Consequently, the observed association between FAI scores and bruxism subtypes may, to some extent, reflect shared method variance or measurement overlap rather than a purely independent clinical relationship. Future studies incorporating clinical examination and instrumental assessments of bruxism are therefore required to clarify the extent to which this association reflects underlying pathophysiological mechanisms.

Although previous literature has suggested that bruxism may influence TMJ pathophysiology through repetitive microtrauma and increased masticatory muscle activity [53, 54], the cross-sectional design of the present study does not permit causal inference. Conversely, TMD-related pain and functional limitations may also influence or exacerbate bruxism behaviors, indicating a potentially bidirectional relationship [51, 52]. Accordingly, these associations should be interpreted with caution, particularly in light of the reliance on self-reported measures without clinical or instrumental confirmation.

Dental students are known to experience elevated levels of stress due to intensive academic demands and clinical responsibilities, and this psychosocial burden has previously been discussed in relation to bruxism-related behaviors [19, 55]. In the present study, most participants reported moderate to high levels of perceived stress. Although no statistically significant differences were observed between bruxism subtypes in terms of categorical stress distribution (Table 3), continuous PSS-10 total scores were higher in the combined bruxism group compared to the AB and non-bruxism groups (Table 4). This apparent discrepancy may be explained by the loss of information associated with categorizing continuous variables, which can reduce statistical power, whereas analysis based on total scores preserves variability and may be more sensitive in detecting between-group differences.

Our present findings suggest that higher perceived stress levels were more frequently observed in individuals with combined bruxism, in whom sleep and awake behaviors co-occur. This pattern may indicate that elevated stress is related to a broader bruxism profile rather than to a single time-specific subtype. Consistent with previous reports indicating mean PSS-10 scores ranging between 18 and 21 in dental students [10, 56, 57], the stress levels observed in the present sample were within the upper range of this distribution, further supporting the view that dental students represent a population exposed to substantial psychosocial burden.

In univariate analyses, higher PSS-10 scores were observed in the combined bruxism group; however, this association did not persist after adjustment for potential confounding factors. In the multinomial logistic regression model, perceived stress (PSS-10) was not independently associated with bruxism subtypes after adjustment for demographic and sleep-related variables. This finding suggests that the associations observed in univariate analyses may partly reflect shared variance with other factors rather than an independent effect of perceived stress. Importantly, multicollinearity diagnostics did not indicate problematic collinearity among the predictors, as tolerance and VIF values remained within acceptable ranges. Therefore, the attenuation of these associations in the multivariable model is more likely to reflect overlapping psychosocial constructs or shared method variance rather than statistical collinearity alone. In addition, BMI categories were re-evaluated in the regression model by combining overweight and obesity due to the small number of participants in the obesity group; however, this modification did not materially alter the regression estimates, suggesting that BMI distribution did not substantially influence the observed associations.

The association between bruxism and psychological factors remains controversial. Although a meta-analysis has suggested an increased likelihood of bruxism in individuals experiencing psychological distress [4], the overall level of evidence has been considered low. While some studies have reported significant associations between stress and bruxism [44, 58], others have not confirmed this relationship [6, 12, 18, 24, 59]. In addition, emotional stress has been suggested to be more strongly associated with AB than with SB [18, 60]. In the present study, the absence of independent associations in multivariable analyses further supports the perspective that stress alone may not be a decisive factor in the expression of bruxism.

Given the multifactorial nature of bruxism, psychological stress is likely to interact with neurophysiological and behavioral mechanisms rather than acting as an isolated determinant [61, 62]. Accordingly, bruxism should be conceptualized within a broader biopsychosocial framework, in which stress may contribute to vulnerability in predisposed individuals but does not independently account for subtype differentiation.

In addition to perceived stress, the present study evaluated sleep quality and insomnia severity. The PSQI, a widely used and validated multidimensional instrument [37, 63], indicated that poor sleep quality was common in this sample, which is consistent with previous reports in dental student populations [22, 24]. In univariate analyses, poorer sleep quality was more prevalent in the combined and SB groups, whereas AB demonstrated a comparatively weaker association with sleep-related parameters. Overall PSQI scores were highest in the combined bruxism group, suggesting a greater burden of sleep-related complaints in individuals exhibiting both sleep and AB behaviors. Similarly, insomnia severity differed across bruxism subtypes. Although most participants did not exceed the threshold for clinical insomnia, higher ISI scores were observed, particularly in the combined and SB groups, whereas AB demonstrated a comparatively weaker profile. These findings suggest that sleep-related forms of bruxism may be more closely associated with subjective sleep disturbances.

Previous literature has suggested that alterations in sleep architecture may be associated with increased central nervous system activity and elevated masticatory muscle tone, which may contribute to bruxism activity [19, 22]. The PSQI evaluates sleep quality across multiple domains, including sleep duration, latency, efficiency, disturbances, medication use, and daytime functioning [36]. Accordingly, higher PSQI scores reflect fragmented and non-restorative sleep, which has been discussed in relation to bruxism-related neuromuscular activity [64].

In the present study, poorer sleep quality was more frequently observed in the combined bruxism group; however, the relatively high prevalence of poor sleep quality in the non-bruxism group suggests that PSQI parameters are not specific to bruxism and may also reflect broader stress-related and lifestyle factors common in student populations. Consistent with this observation, multinomial logistic regression analyses indicated that neither PSQI nor ISI remained independently associated with bruxism subtypes after adjustment for demographic and other covariates. These findings suggest that the observed between-group differences in sleep parameters may reflect shared associations with other variables rather than independent predictive effects. Overall, these results support an association between bruxism, particularly combined and SB, and poorer self-reported sleep health; however, given the cross-sectional design and reliance on self-reported measures, no conclusions can be drawn regarding directionality or causality.

The literature remains inconsistent regarding the relationship between sleep quality and bruxism. While some studies have reported associations between PSQI scores and SB [63, 65], others have not confirmed such findings [43, 66]. A recent systematic review identified associations for possible and probable SB, but not for definite bruxism assessed using instrumental methods [17]. Similarly, meta-analytic data suggest that associations are more consistently observed in self-report-based studies, whereas findings are less consistent when objective measures such as polysomnography are employed [16]. These discrepancies highlight the influence of methodological variability and the inherent limitations of self-reported sleep assessment.

These findings further emphasize the complex relationship between bruxism, sleep quality, and insomnia. Although insomnia scores were higher in the sleep and combined bruxism groups in univariate analyses, multivariable regression analyses did not identify insomnia severity as an independent predictor of bruxism subtypes, suggesting that the observed differences may reflect shared underlying factors rather than a direct or isolated effect of insomnia.

Previous studies have reported associations between self-reported insomnia complaints and SB [67, 68, 69], whereas findings based on objective assessments have been less consistent. Neurophysiological mechanisms, including alterations in dopaminergic and serotonergic pathways as well as increased sympathetic activation, have been proposed to explain potential links between sleep fragmentation and masticatory muscle activity [64, 70]. However, given the cross-sectional design and reliance on self-reported measures, the present findings should be interpreted cautiously.

From a clinical perspective, these results suggest that assessment of sleep-related complaints may be relevant when evaluating bruxism in young adult populations. At the same time, sleep disturbances appear to operate within a broader biopsychosocial context rather than as independent determinants of bruxism.

Self-reported bruxism was found to be highly prevalent among dental students, with the combined subtype demonstrating a greater self-reported TMD symptom burden and more pronounced sleep-related complaints in univariate analyses. However, after multivariable adjustment for sex, smoking status, BMI, academic year, perceived stress, sleep quality, and insomnia severity, only self-reported TMD symptom severity (FAI) remained independently associated with bruxism subtypes. Neither perceived stress, sleep parameters, nor demographic variables demonstrated independent predictive value in the adjusted models. In this context, self-reported TMD symptom severity emerged as the variable most consistently associated with bruxism subtypes in this cohort, and the consistently higher symptom burden observed in the combined subtype suggests that the coexistence of sleep and AB behaviors may represent a subgroup with greater overall self-reported symptom burden.

Overall, these findings indicate that although psychosocial stress and sleep disturbances frequently co-occur with bruxism, particularly in the combined subtype, their associations may not be independent when potential confounding factors are considered. The results underscore the importance of evaluating bruxism subtypes separately and of integrating the assessment of TMD-related symptoms into the clinical evaluation of young adults exposed to academic stress. Nevertheless, given the cross-sectional design and reliance on self-reported measures, causal inferences cannot be established. In addition, these findings support a shift away from a purely occlusal perspective and highlight the relevance of adopting a biopsychosocial approach in the evaluation of bruxism in young adult populations. Future longitudinal studies incorporating clinical examination and instrumental confirmation are required to clarify the directionality and underlying mechanisms of these relationships.

## 5. Limitations

This study has several limitations. First, the cross-sectional design only allowed assessment of associations between bruxism, stress, sleep quality, insomnia, and TMD symptoms at a single time point, and therefore does not allow conclusions about causality. In addition, bruxism was assessed only based on self-reported (subject-based) measures. Objective methods such as clinical examination, polysomnography, or surface electromyography were not used. Furthermore, specific AB behaviors such as mandibular thrusting and sustained jaw bracing were not evaluated. As a result, AB may have been underestimated, and the variability within this subgroup could not be fully captured. This may also have affected the distribution of bruxism subtypes, possibly contributing to a higher proportion of combined bruxism in this sample. Because the data were self-reported, recall bias and misclassification cannot be excluded. In particular, the use of self-reported possible bruxism may have led to overestimation of prevalence compared to that in studies using clinical or instrumental confirmation.

The study sample was obtained from a single dental school using convenience sampling, which may limit generalizability to other populations. The predominance of female participants may also have influenced the results. In addition, stress, sleep quality, insomnia, and TMD symptoms were all assessed using self-report instruments, which may reduce objectivity and introduce common method bias.

## 6. Conclusions

This study showed that combined bruxism was associated with higher self-reported TMD symptom levels and more pronounced sleep-related complaints in univariate analyses. However, after adjusting for sex, smoking status, BMI, academic year, perceived stress, sleep quality, and insomnia severity, only TMD symptom severity (FAI) remained independently associated with bruxism subtypes. Perceived stress and sleep-related parameters were not independent predictors in the adjusted model. These results suggest that although stress and sleep problems often occur together with bruxism, especially in the combined subtype, their relationships may not be independent when other factors are considered. In contrast, TMD symptom severity appears to be the factor most consistently associated with bruxism subtypes in this population. From a clinical perspective, these findings support the importance of assessing TMD symptoms when evaluating bruxism in young adults. At the same time, bruxism should be considered within a broader biopsychosocial context rather than only from an occlusal perspective. Future longitudinal studies with clinical and instrumental assessment are needed to better understand the direction and mechanisms of these relationships.

## Supplementary Material

Supplementary material associated with this article can be found, in the online version, at https://files.jofph.com/files/article/2075480929344602112/attachment/Supplementary%20material.docx.


