# Supplementary material for: Biopsychosocial assessment of temporomandibular disorder symptoms across bruxism subtypes in dental students: associations with stress, sleep quality, and insomnia
Source: J Oral Facial Pain Headache. 2026 Jul 12;40(4):130–44. doi: 10.22514/jofph.2026.055 (PMC13434143; doi:10.22514/jofph.2026.055)
Supplement: Supplementary file 1 [file Supplementary-material.docx]

Supplementary material

# Supplementary File 1. Self-report questionnaire for the identification of possible combined, sleep and awake bruxism

This questionnaire was adapted from the diagnostic approach proposed by Phuong *et al.* [1] (2020), based on the recommendations of the American Academy of Sleep Medicine [2] and the questions developed by Pintado *et al.* [3] (1997). It was used in the present study to identify possible bruxers through self-reported data.

**Possible Sleep Bruxism (SB):**

1. Are you aware, or has anyone heard you, grinding your teeth frequently during sleep?

2. Are you aware that your dentition is worn down more than it should be?

3. Are you aware of any of the following symptoms upon awakening?

4. (a) Fatigue, tightness, or soreness of your jaw.

5. (b) Feeling that your teeth are clenched or that your mouth is sore.

6. (c) Aching of your temples.

7. (d) Difficulty in opening your mouth wide.

8. (e) Tension or stiffness in your jaw joint, requiring movement to release.

9. (f) Clicking in your jaw joint that disappears afterward.

**Possible Awake Bruxism (AB):**

4. Are you ever aware of grinding your teeth during the day?

5. Are you ever aware of clenching your teeth during the day?

**Classification Criteria:**

● Participants answering “Yes” to Q1 and/or Q2, or to at least one symptom in Q3, were classified as possible sleep bruxers (SB).

● Participants answering “Yes” to Q4 and/or Q5 were classified as possible awake bruxers (AB).

● Participants answering “Yes” to at least one sleep-bruxism question (Q1–Q3) and at least one awake-bruxism question (Q4–Q5) were classified as combined bruxers (CB).

● Participants with all “No” responses were classified as non-bruxers (NB).

References

[1] Phuong NTT, Ngoc VTN, Linh LM, Duc NM, Tra NT, Anh LQ. Bruxism, related factors and oral health-related quality of life among Vietnamese medical students. International Journal of Environmental Research and Public Health. 2020; 17: 7408.

[2] Sateia MJ. International classification of sleep disorders-third edition: highlights and modifications. Chest. 2014; 146: 1387–1394.

[3] Pintado MR, Anderson GC, DeLong R, Douglas WH. Variation in tooth wear in young adults over a two-year period. Journal of Prosthetic Dentistry. 1997; 77: 313–320.
